# Supplementary material for: Patterns of Intron Gain and Loss in Fungi
Source: PLoS Biol. 2004 Nov 30;2(12):e422. doi: 10.1371/journal.pbio.0020422 (PMC532390; doi:10.1371/journal.pbio.0020422)
Supplement: Table S1 — Also available at http://genes.mit.edu/NielsenEtAl/. (4.3 MB ZIP). [file pbio.0020422.st001.zip › NielsenEtAl/html/33.html]

AN4019.1.NCU03107.1.MG03701.1.FG00924.1


```
 CLUSTAL W (1.82) Multiple Sequence Alignments - Introns Inserted


Sequence 1: MG03701.1	587 aa
Sequence 2: FG00924.1	452 aa
Sequence 3: NCU03107.1	568 aa
Sequence 4: AN4019.1	494 aa
Alignment Length: 609 aa
Number Identitical Residues: 132 aa
Alignment Score (without introns) 8691


MG03701.1 	MPFISSACTSAVLPTVFACCMGLFIQLKLVDVGSLNELAWEPKTAAET1VARFAEPLALT
NCU03107.1	----------------------------MVTKNKDGPKLPKQQLAILA1IARFAEPLAAT
FG00924.1 	------------------------------------------------~-----------
AN4019.1  	------------------------------------------------~-----------
          	                                                            

MG03701.1 	SVFPYLPEMIASFGVDKDSIAKWAGLTGATFSVCQSVFAVPWGLLSDRVGRKPTILFGLV
NCU03107.1	SIYPYLPEMIKNFGVPKNEVAKWAGFTGSIFSVAQSMAAVPWGRASDRFGRKPIILLGIT
FG00924.1 	--------------------------------------------------------MGLL
AN4019.1  	--------MIESVGVPQSEVAKWAGLTSAISSFSQAAMAVYWGTASDRFGRKPIILLGLT
          	           . .  ... :. :. :.:  : ..:  :   .  :.  . ..   :*: 

MG03701.1 	1STMICFLIWGMSTTLAMAMTIRAVQGASNGNV~GIIRTMVAEMVTDKEHQPRAFSIM0P
NCU03107.1	~STMICFILWGLSTSLTMAITVRFIMGAGNGNV1GILRTMVAEMVPEKELQPKAFSLM~P
FG00924.1 	~TTMVCFIVWGMSTSLPMAIVVRAVQGGGNGNV1GIIRTMVAEMVPERELQPRAFSIM~P
AN4019.1  	~ATMVLSLAFGLSKSLPMLITCRGMIGFMNGNV~GIIRTMVAEMVQDKELQPRAFSIM~P
          	 :**:  : :*:*.:*.* :. * : *  **** **:******** ::* **:***:* *

MG03701.1 	LVWSIGSVFGPAFGGFFARPAEQFPSLFGRIEFFKRYPFALPNIIACFFFLVSVITGTLF
NCU03107.1	LVWSIGSVFGPAFGGFFAQPAKQYPSLFGNIDFFKKYPFALPNIVAGIIFFISLMTGLLF
FG00924.1 	IVWSLGSIIGPSFGGFFAEPAEQYPEIFGHMEFFKRFPFILPNLILTVFFLTSVTVAVLF
AN4019.1  	MVWTIGSIFGPSFGGSLARPTEKYPEIFGHSWFFKEYPFVLPNMVAGFFFIIGISTGFLF
          	:**::**::**:*** :*.*::::*.:**.  ***.:** ***::  .:*: .: .. **

MG03701.1 	LK0ETLASKRDTEDWGLKLGEKLTRPFK------SRK--GPQHRPSFVDGEATAPLLPRP
NCU03107.1	LR~ETLESRRGHQDWGLALGEKLTRPFKRRGPHYHHHHDRNNRRHSFVDDGASAPLLAPS
FG00924.1 	LH0ETLPSKRGHRDWGILVGERITRSFK------NNRPMPSTRRPSFVDGEATSPLLPNK
AN4019.1  	LH0ETLHTKQGYRDSGLVLGQMLTGLCTG----------NCRKVTKRLEDDETTPLLG-E
          	*: *** :::. .* *: :*: :*   .              :  . ::.  ::***   

MG03701.1 	AAAEPVNTPTAPEPKAKIRDIFTYATVINLICYTFLAMHSVAFDQLLPVFLHHP---HQK
NCU03107.1	SLPTSIAS--VETDPPTLREIFTPQTIINLTSYTFLALHSVTFDQVLPVFLNYPRVSPHD
FG00924.1 	IAPKTHSQ----EPARKER-VLTRETSINLLAYTFLAFHSVAYDQILSVFLRHP---VEE
AN4019.1  	RLPASKHQIKAEVKKHSWREVLNPQSVLILLAYTLMSVHTMAFESVLPVFLHTP--VQHL
          	  . .    .      . *.::.  : : * .**:::.*:::::.:*.***. *    . 

MG03701.1 	STPENTSLPFRFSSGFGLS~---SDKIGAIFMVYGIVCG0LIQFVM~FPPLCNRFGVLRC
NCU03107.1	RTPSNTHLPFKFSGGFGLS~---SDKIGTIYTVYGIACG~VVQFFL~FPWFCARFGVLRC
FG00924.1 	HTPENTAFPFYFSGGFGMS~---HSQVGLIYTVYGVVCG~TIQFTL2F------------
AN4019.1  	QDNPDVQLPFKFVGGFGMV1PSDSQRIGFFYTITGCIGI~VMQFYV~FPFCAKRFGVLNC
          	    :. :** * .***:  .:. .::* :: : *      :** : *.  .   .   .

MG03701.1 	YRAAA1LMFPVCYFLTPFTSLVHGTYGRYIALMFIMLLKGFAVIIGFPCLTIMLTNSASS
NCU03107.1	FRAAT~LLFPIVYLLTPYTALIEDTHTRYAVFLTLILVKGVAVIIGFPCTTILLTNSASS
FG00924.1 	---CC1ILMPIAYFLTPYCVLFSTHNARTTALILVMFIKAAGIIVAFPSTTILLTNSCSS
AN4019.1  	VKASA~AVFPIIYLLTPYIALVPES-LRSISICLLILSKLTASIFNFPGITILLTNSAGS
          	  :.   ::*: *:***:  *.     *   :  ::: *  . *. **  **:****..*

MG03701.1 	LSILGTLNGIATTTSGFGRAVGPASAGAIFSWGIQ-RGYVISGWWFLGTVAVVGAIPPFF
NCU03107.1	LRILGTLNGFATTFSGLGRAIGPAMAGAIFTWGVTGRDYIIAPWWFLSLVAAIGAIPSWW
FG00924.1 	LRVLGTLNGYATTFSGLGRATGPASAGALFTWGAE-HGYVVTAWFFLMFVAILGAIPAYL
AN4019.1  	LSILGTLNGVATSMSAVGRAAGPAMLGPIFSLGLR-AGFIILPWWFLCAISALAATPILW
          	* :****** **: *..*** ***  *.:*: *    .:::  *:**  :: :.* *   

MG03701.1 	ITEGSGPYADAQGDETDIDEDTVAEADPIPIPESTAGPLGPHEAGFPSPPVPSESSRLRG
NCU03107.1	IVEGEGPTRSLQTDSEESGEEEGEEEED----EEEGVEVLDEGSTLVSNSQGSRAS----
FG00924.1 	AKDGAGPTASASTSAENSDTEDNEASS------SSSTLLLPENSAVASDSEDEEQPLNKS
AN4019.1  	IVERDEFQDQDMGQSESSDESETEAER---------GTVHPAKSQTRTES----------
          	  :      .   .  . . .                 :    :   : .          

MG03701.1 	RSLPKSYGTTQ-------
NCU03107.1	STMDGGYGTIKKDAQEAR
FG00924.1 	RPVQQSYGTIKGGRN---
AN4019.1  	-----NHGT---------
          	     .:**
```
